# Case Report: Conversion from VA-ECMO to A-NRP for organ donation in a patient in asystole with Stanford A aortic dissection

**DOI:** 10.3389/fcvm.2026.1733614

**Published:** 2026-03-02

**Authors:** Benjamin Assouline, Franz Immer, Philippe Compagnon, Charles-Henri Wassmer, Hervé Quintard, Giorgios Giannakopoulos, Birgit Andrea Gartner, Karim Bendjelid, Raphaël Giraud

**Affiliations:** 1 Intensive Care Division, Department of Acute Care Medicine, Geneva University Hospitals, Geneva, Switzerland; 2 Faculty of Medicine, University of Geneva, Geneva, Switzerland; 3 Department of Anesthesiology, Pharmacology, Intensive Care and Emergency Medicine, Geneva Hemodynamic Research Group, Geneva, Switzerland; 4 Swisstransplant, Bern, Switzerland; 5 Division of Transplantation, Department of Surgery, Geneva University Hospitals, Geneva, Switzerland; 6 Division of Cardiology, Department of Medicine, Geneva University Hospitals, Geneva, Switzerland; 7 Division of Emergency Medicine, Department of Acute Care Medicine, Geneva University Hospitals, Geneva, Switzerland

**Keywords:** aortic dissection, ECPR, normothermic regional perfusion, organ procurement, organ transplantation, refractory cardiac arrest, VA-ECMO

## Abstract

Survival after cardiac arrest remains poor, with fewer than 10% of patients surviving out-of-hospital cardiac arrest. Extracorporeal cardiopulmonary resuscitation (ECPR) has emerged as a strategy to address this unmet clinical need. Although ECPR is associated with improved survival and neurological outcomes compared to conventional care, a significant proportion of patients still present with irreversible anoxic brain injury. In that context, organ donation after circulatory death (DCD) may be proposed to families and represents an important opportunity to expand the donor pool. We report the case of a 63-year-old patient who presented with cardiac arrest secondary to a Stanford A type aortic root dissection. Following ECPR, the patient remained in persistent asystole. While supported on veno-arterial extracorporeal membrane oxygenation, he subsequently developed massive hemoptysis in the context of left atrial, ventricular, and pulmonary venous thrombosis. An emergency DCD procedure with abdominal normothermic regional perfusion (A-NRP) was performed, enabling successful kidney procurement and transplantation. Complete exclusion of the descending aorta using an aortic occlusion balloon resulted in immediate cessation of hemoptysis. This case illustrates an exceptional scenario in which ECPR performed in the setting of Stanford type A aortic dissection resulted in persistent asystole, complete left-sided cardiac, and massive hemoptysis. Given the confirmed irreversible prognosis and the previously expressed wish of the patient to donate organs, an emergency DCD procedure with A-NRP was the only viable strategy, ultimately allowing successful procurement and transplantation of one kidney.

## Introduction

Survival after cardiac arrest (CA) remains dismal, with fewer than 10% of patients surviving out-of-hospital cardiac arrest (OHCA). Extracorporeal cardiopulmonary resuscitation (ECPR) has been implemented to address this unmet clinical need. Although ECPR is associated with improved survival and neurological outcomes compared with conventional care (1), many patients will still sustain irreversible anoxic brain injury. In such cases, organ donation after circulatory death (DCD) may be proposed to families and represents an important opportunity to expand the donor pool (2). Notably, DCD with abdominal normothermic regional perfusion (A-NRP) has demonstrated significant benefits for liver and kidney graft outcomes and is increasingly being adopted (3).

## Case description

A 63-year-old man with no significant past medical history suddenly developed acute chest pain radiating to the jaw, prompting an immediate call to emergency medical services. Upon arrival of paramedics, he was initially alert but rapidly deteriorated into CA with pulseless electrical activity (PEA). Immediate cardiopulmonary resuscitation (CPR) was initiated, with a no-flow time of 0 min, supported by a medical mobile unit dispatched to the scene. Despite 15 min of advanced life support (ALS), including endotracheal intubation and epinephrine administration, there was no return of spontaneous circulation (ROSC). In accordance with our local protocol, the patient was transferred to our center under ongoing CPR for ECPR evaluation.

**Figure 1:**
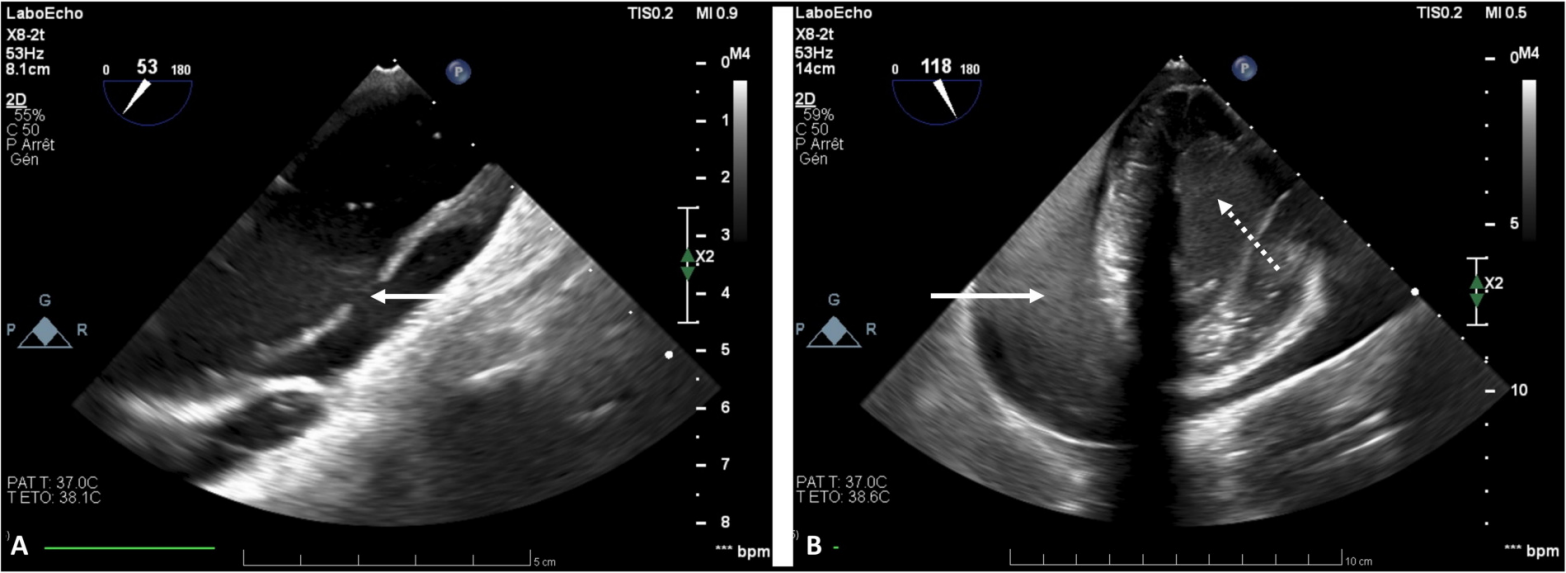
Transesophageal echocardiography while on VA-ECMO support showing Stanford A type aortic dissection of the ascending aorta [ **(A)** white solid line arrow], cardiac tamponade [ **(B)** white solid line arrow], and spontaneous echo contrast in the left ventricle [ **(B)** white dotted line arrow].

Upon arrival in the emergency department, ALS was continued, and a point-of-care ultrasound (POCUS) revealed a small pericardial effusion, which, in the context of OHCA, was interpreted as a non-specific periarrest finding compatible with several differential diagnoses, including acute coronary syndrome with possible free-wall rupture, postresuscitation myocardial dysfunction, or non-specific effusion. Importantly, no sonographic feature suggestive of an aortic dissection (no visible flap, no aortic root enlargement, no severe aortic regurgitation) were identified. An arterial blood gas analysis revealed severe acidosis (pH 7.11), elevated PaCO₂ (9.8 kPa), and markedly increased lactate levels (9.7 mmol/L). After 22 min of low-flow time, and despite the presence of a non-shockable rhythm, the multidisciplinary team decided to proceed with ECPR, and veno-arterial extracorporeal membrane oxygenation (VA-ECMO) was initiated according to institutional protocol (total low-flow time = 39 min). Transesophageal echocardiography (TEE) was initially performed to confirm correct cannula placement. Once VA-ECMO flow restored sufficient hemodynamic stability to allow a comprehensive examination, TEE subsequently revealed a Stanford type A aortic dissection extending from the aortic root to the arch, without involvement of the supra-aortic vessels (Figure 1), along with a progressively enlarging pericardial effusion.

**Figure 2:**
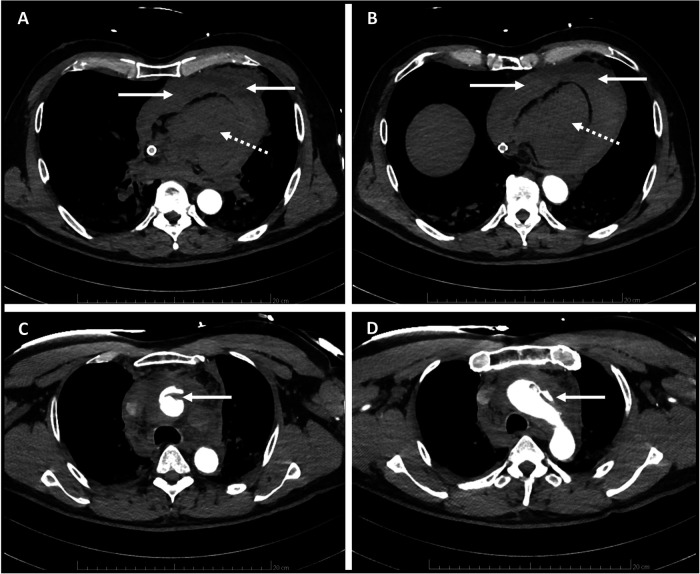
Contrast-enhanced CT scan showing **(A,B)** *no* enhancement of *cardiac chambers (*white dotted line arrow) and cardiac tamponade (white solid line arrow) and **(C,D)** Stanford A type aortic dissection of the ascending aorta and aortic arch (white solid line arrow).

A contrast-enhanced CT scan confirmed rupture at the aortic root with extensive hemopericardium (Figure 2). The Stanford type A dissection was confined to the ascending aorta and aortic arch, without extension into the abdominal aorta or femoral bifurcation, which enabled successful implantation of VA-ECMO.

Cardiothoracic surgery was consulted and concluded that prolonged myocardial ischemia (>2 h) and refractory CA contraindicated surgical intervention. Before admission to the intensive care unit (ICU), the family of the patient met with the clinical team and were informed that the prognosis was overwhelmingly poor, with a very high likelihood of irreversible neurological injury given the prolonged low-flow time and absence of cardiac activity. However, the team also explained that, in exceptionally rare circumstances, early reperfusion might allow limited neurological recovery and, in the absence of sequelae, could even lead to candidacy for heart transplantation. For these reasons, it was deemed essential to allow sufficient time to assess the neurological evolution before any definitive decision regarding withdrawal of the treatment. During this discussion, the spouse of the patient stated that he had previously expressed a clear willingness to donate his organs in the event of treatment withdrawal or progression to brain death. This information guided subsequent ethical and clinical decision-making. The patient was then admitted to the ICU, and sedation (consisting of a total of 10 mg IV midazolam) was discontinued immediately upon arrival.

No systemic anticoagulation was initiated because initial coagulation studies revealed profound postcardiac arrest coagulopathy (Quick 47%, INR 1.51, PTT 81.7 s, fibrinogen 2.0 g/L). The ECMO circuit and cannulas were heparin-coated, and ECMO flow was maintained at up to 4 L/min. After 12 h of ICU management on VA-ECMO, the patient achieved hemodynamic stability with low-dose norepinephrine (0.12 µg/kg/min) and fluid resuscitation exceeding 4 L. He remained in asystole, and echocardiography demonstrated complete cardiac thrombosis with absence of pulmonary blood flow.

Despite the presence of spontaneous breathing, the Glasgow Coma Scale score remained 3. EEG revealed a symmetrical low-voltage theta background with absent reactivity, no posterior dominant rhythm, and no burst-suppression pattern, findings consistent with a highly malignant pattern indicative of severe encephalopathy.

Following a discussion with the family regarding the poor neurological prognosis, the spouse of the patient confirmed that he would not have wished to survive with any neurological sequelae and expressed his prior willingness to be an organ donor. Terminal analgosedation was initiated, and a decision was made to proceed with organ DCD using A-NRP.

The procedure was initially planned for the following morning. However, the patient developed massive hemoptysis. Despite optimization of mechanical ventilation settings, the bleeding remained uncontrollable, prompting advancement of the DCD procedure. Withdrawal of life-sustaining therapy was undertaken, and ECMO was discontinued, rapidly leading to the death of the patient. The ECMO circuit was disconnected, the cannulas purged and placed in short-circuit mode. Following a 5-min no-touch period, death was formally declared after neurological examination, in accordance with the recommendations of the Swiss Academy of Medical Sciences. Subsequently, the left femoral arterial catheter was exchanged for an 11-Fr introducer. Under subxiphoid echocardiographic guidance, a Medtronic RELIANT aortic occlusion balloon was advanced to a supradiaphragmatic position and inflated with 25 mL of saline. The femoral ECMO cannulas were then reconnected to the A-NRP circuit, which was initiated with a blood flow of 3.5 L/min. The balloon achieved complete thoracic isolation, as evidenced by supradiaphragmatic mottling and, notably, complete cessation of hemoptysis.

**Figure 3:**
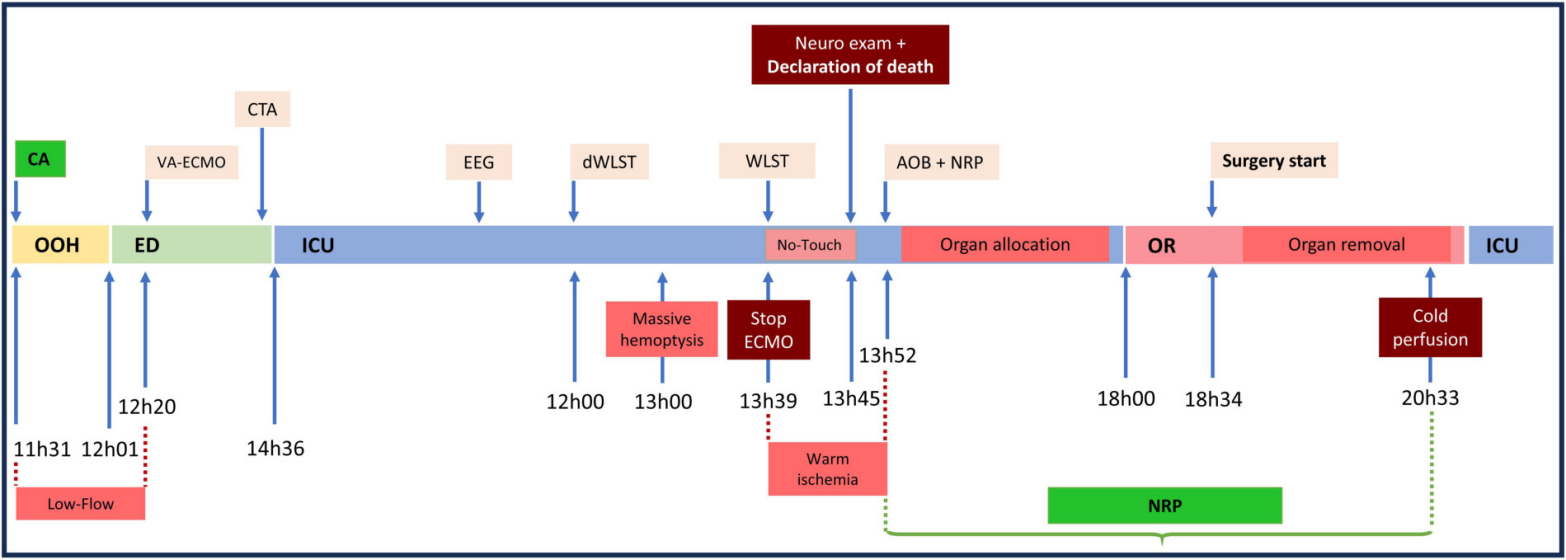
Timeline of clinical events from cardiac arrest to organ procurement. Timeline summarizing key clinical, diagnostic, and procedural events from out-of-hospital cardiac arrest to organ procurement, including VA-ECMO initiation, WLST decision, declaration of death, aortic occlusion balloon insertion with NRP initiation, transfer to the OR, surgery, and organ removal. CA, cardiac arrest; OOH, out-of-hospital; ED, emergency department; ICU, intensive care unit; CTA, computed tomography angiography; VA-ECMO, veno-arterial extracorporeal membrane oxygenation; EEG, electroencephalogram; dWLST, decision of withdrawal of life-sustaining therapy; WLST, withdrawal of life-sustaining therapy; AOB, aortic occlusion balloon; NRP, normothermic regional perfusion; OR, operating room.

The interval from brain death declaration to initiation of A-NRP was 6 min, the abdominal warm ischemia time was 13 min, and A-NRP was maintained for 401 min to allow organ allocation and procurement. Hemodynamic support included administration of 2 L of 0.9% NaCl, transfusion of two units of packed red blood cells, and norepinephrine at 0.12 µg/kg/min to maintain a mean arterial pressure above 60 mmHg, monitored via a left femoral sheath. Ultimately, the right kidney was procured and transplanted. The recipient required 1 week of postoperative dialysis, after which renal function progressively recovered. At present, graft function is satisfactory, dialysis has been discontinued, and the recipient has been discharged home for several weeks. The timeline of clinical, diagnostic, and procedural events from OHCA to organ procurement is presented in Figure 3.

## Discussion

Given the discrepancy between the number of patients awaiting organ transplantation and the limited number of donors, all efforts must be made to expand the donor pool (4). In 2024, Switzerland had 2,116 patients on the transplant waiting list, while only 187 deceased organ donors were registered, resulting in 637 transplants, including 115 from living donors. Currently, two types of organ donation are recognized: donation after brain death (DBD) and DCD (Maastricht III). In the case of DCD, two organ procurement strategies are used: super-rapid recovery (SRR) and NRP (5). A-NRP consists of establishing femoro-femoral VA-ECMO with placement of an occlusion balloon in the descending aorta, allowing reperfusion of the abdominal organs while preventing reperfusion of the heart and brain (6). This technique has demonstrated improvement in the outcome of grafts and transplanted patients compared to SRR. Moreover, the use of A-NRP appears to increase utilization rates of abdominal organs, particularly kidneys (3, 7–10). Therefore, A-NRP has become mandatory for liver procurement in several European countries. In addition, as reported recently, A-NRP can allow the excision and pathological examination of an abdominal mass, while perfusing the abdominal organs before allocation and organ procurement (11).

Refractory CA is associated with poor outcomes (12), even in patients supported with ECPR. In this situation, organ donation should be considered (13). In fact, a secondary analysis of a Prague CA randomized trial showed that ECPR was associated with an increased rate of organ donation and excellent outcomes of transplanted organs (14). In the present case, aortic dissection was not suspected prior to cannulation. The small pericardial effusion identified on POCUS was considered non-specific and compatible with several other peri-CA etiologies. Full diagnosis only became possible after VA-ECMO implantation, when TEE and subsequent CT could be completed under stable hemodynamic conditions. Once the diagnosis of Stanford type A dissection was established, VA-ECMO was temporarily continued to allow full evaluation of surgical feasibility, neurological potential, and, critically, to inform the family and clarify the patient's previously expressed wishes regarding organ donation. Discontinuation of ECMO at this stage would have precluded these essential steps.

Although the patient ultimately presented irreversible cardiac and neurological failure, established evidence from cohort studies and systematic reviews shows that a subset of patients treated with ECPR after initially refractory cardiac arrest can achieve meaningful neurological recovery (15, 16). Furthermore, heart transplantation has been reported in patients with prior Stanford type A dissection in selected and highly specific contexts (17), although not in the setting of ECPR for dissection-related cardiac arrest. Therefore, while exceedingly unlikely in this case, a purely theoretical scenario in which early reperfusion and limited aortic involvement might have allowed some degree of neurological recovery, and thereby preserved, in principle, the possibility of future transplant candidacy, cannot be entirely excluded. This rationale further supports the decision to maintain ECMO briefly after diagnosis rather than discontinue support immediately.

In the present case, the heart of the patient remained in asystole despite VA-ECMO support and rapidly developed massive hemoptysis. The mechanism by which the aortic occlusion balloon abruptly halted the hemoptysis warrants specific attention. In the absence of native cardiac contraction and in the presence of complete thrombosis of the left atrium, ventricle, and pulmonary veins, pulmonary venous drainage was entirely obstructed, resulting in profound pulmonary congestion. Under VA-ECMO support, systemic flow continued to perfuse the bronchial arterial circulation through branches of the descending thoracic aorta. Although physiologically representing a minor fraction of cardiac output, this systemic inflow can become the predominant source of pulmonary perfusion when pulmonary arterial flow is absent and venous outflow is obstructed. In this setting, elevated bronchial arterial pressure combined with severe venous congestion likely led to capillary rupture and massive hemoptysis. Advancement and inflation of the aortic occlusion balloon interrupted all systemic arterial inflow to the thorax, thereby suppressing bronchial circulation flow and abolishing ECMO-driven retrograde pressure transmission to the pulmonary microvasculature. This thoracic isolation eliminated the pressure gradient across the pulmonary capillary bed, leading to immediate cessation of hemoptysis. To respect the prior wish of the patient to donate organs and with the agreement of the family, the DCD procedure was carried out emergently. Following initiation of A-NRP, abdominal perfusion was safely established for organ retrieval. The procured kidney was successfully transplanted, with immediate graft reperfusion and no delayed graft function reported in early postoperative follow-up.

The absence of systemic heparinization during the early VA-ECMO phase reflected both the structural risk of exacerbating a ruptured Stanford type A dissection with hemopericardium and the presence of profound coagulopathy at admission. Circuit thrombosis did not occur, likely due to high ECMO flow and heparin-coated components. The subsequent intracardiac thrombosis was therefore attributed to the complete absence of cardiac ejection rather than to the lack of anticoagulation.

Left ventricular (LV) overload is a well-recognized and challenging complication of VA-ECMO support and can contribute to pulmonary edema and intracardiac thrombosis when unloading is inadequate (18). Several unloading strategies have been proposed, including the use of inotropes, intra-aortic balloon counterpulsation, and microaxial flow pumps such as the Impella CP (19). However, none of these approaches were applicable in the present case, as the patient exhibited complete absence of cardiac contraction with total thrombosis of the left-sided chambers, making mechanical or surgical unloading both physiologically impossible and potentially harmful. Moreover, in the context of imminent organ donation, any intervention risking thromboembolism to the abdominal organs was contraindicated. This case highlights not only the physiological mechanisms underlying hemoptysis control but also the unique procedural feasibility related to the specific anatomy of the patient: because the dissection did not extend beyond the aortic arch, femoral cannulation and subsequent A-NRP were technically feasible. Most TAAD cases involve the descending aorta or iliac arteries, which would preclude such an approach.

This case has several limitations. First, this case involved an atypical Stanford type A dissection pattern that, unlike most cases, permitted safe femoral cannulation and initiation of A-NRP. Second, the success of organ donation relied on an extremely rapid prehospital response and immediate access to ECPR, conditions that are not always available. Third, the risk of multiorgan dysfunction following dissection-related cardiac arrest is substantial and often compromises organ viability, limiting the generalizability of the favorable organ outcomes observed in this case. Finally, as with all single-case reports, the ability to extrapolate these findings to broader clinical practice is inherently limited. These factors should be carefully considered when interpreting this case.

## Conclusion

This case report illustrates ECPR failure in the context of Stanford type A aortic dissection, complicated by persistent asystole, left heart thrombosis, massive hemoptysis, and a poor neurological prognosis in a patient who had expressed a wish to donate organs. In this setting, emergency DCD with A-NRP represented the only viable strategy to control massive hemoptysis, preserve abdominal organ perfusion, and enable successful transplantation.

## Data Availability

The raw data supporting the conclusions of this article will be made available by the authors, without undue reservation.
